# Mood Induction Differently Affects Early Neural Correlates of Evaluative Word Processing in L1 and L2

**DOI:** 10.3389/fpsyg.2020.588902

**Published:** 2021-01-12

**Authors:** Johanna Kissler, Katarzyna Bromberek-Dyzman

**Affiliations:** ^1^Department of Psychology, Bielefeld University, Bielefeld, Germany; ^2^Faculty of English, Adam Mickiewicz University, Poznań, Poland

**Keywords:** mood, emotion, language, bilingualism, word processing, context

## Abstract

We investigate how mood inductions impact the neural processing of emotional adjectives in one’s first language (L1) and a formally acquired second language (L2). Twenty-three student participants took part in an EEG experiment with two separate sessions. Happy or sad mood inductions were followed by series of individually presented positive, negative, or neutral adjectives in L1 (German) or L2 (English) and evaluative decisions had to be performed. Visual event-related potentials elicited during word processing were analyzed during N1 (125–200 ms), Early Posterior Negativities (EPN, 200–300 ms and 300–400 ms), N400 (350–450 ms), and the Late Positive Potential (LPP, 500–700 ms). Mood induction differentially impacted word processing already on the N1, with stronger left lateralization following happy than sad mood induction in L1, but not in L2. Moreover, regardless of language, early valence modulation was found following happy but not sad mood induction. Over occipital areas, happy mood elicited larger amplitudes of the mood-congruent positive words, whereas over temporal areas mood-incongruent negative words had higher amplitudes. In the EPN-windows, effects of mood and valence largely persisted, albeit with no difference between L1 and L2. N400 amplitude was larger for L2 than for L1. On the LPP, mood-incongruent adjectives elicited larger amplitudes than mood-congruent ones. Results reveal a remarkably early valence-general effect of mood induction on cortical processing, in line with previous reports of N1 as a first marker of contextual integration. Interestingly, this effect differed between L1 and L2. Moreover, mood-congruent effects were found in perceptual processing and mood-incongruent ERP amplification in higher-order evaluative stages.

## Introduction

Bilinguals use two language systems to communicate and comprehend emotional meanings. Previous research has pointed to both differences and similarities in sensitivity to emotional content in bilinguals when they operate in their L1 and L2 (e.g., Pavlenko, 2012; Caldwell-Harris, 2015). It has indicated that linguistic systems acquired at different stages in life and with different proficiency, may vary also in the degree and depth of affective integration. Importantly, words people use to share meanings come coupled with contextual embeddings. Situational, social and/or emotional contexts may endow single words’ meanings with personal relevance, or communicative salience, and thus modify their processing. Transient feelings—moods, constitute one such communicative embedding: an emotional context against which words meanings are comprehended and interpreted. Here, we investigate whether and how moods modify the neurophysiological dynamics of word processing in the two linguistic systems of German-English bilinguals: German (L1) and English (L2).

Research on neural correlates of emotional word processing in L1 shows that valenced words, i.e., positive and negative ones, are processed more rapidly and evoke larger responses than neutral words (for reviews see, Kissler et al., 2006; Citron, 2012; Hinojosa et al., 2019). Event-related potentials (ERPs) research has shown that emotional features of words influence brain signatures at temporally distinct ERP components (e.g., Kissler and Herbert, 2013). Emotion effects for words have been most consistently reported at the early posterior negativity (EPN), peaking at around 200–300 ms post-stimulus, demonstrating higher amplitudes for emotional rather than neutral words (e.g., Kissler et al., 2007, 2009; Herbert et al., 2008; Palazova et al., 2011, 2013; Citron et al., 2013). N400 amplitudes, peaking around 400 ms, and showing smaller amplitudes for emotional than for valence-free words are also often reported (e.g., Sass et al., 2010; Palazova et al., 2013; Zhang et al., 2014). At later, integration stages emotional words typically elicit enhanced late positive potential (LPP) amplitudes peaking between 400 and 800 ms (e.g., Herbert et al., 2006, 2008; Hofmann et al., 2009; Schacht and Sommer, 2009a; Kissler and Herbert, 2013). By contrast, emotion effects at very early temporal stages are more sporadically observed (cf., Citron, 2012). Studies that detected such early effects, report amplified amplitudes on P1, peaking between 80 and 120 ms (e.g., Hofmann et al., 2009; Scott et al., 2009; Bayer et al., 2012; Schindler et al., 2019b), and/or on N1 between 100 and 200 ms post stimulus. They are typically valence-specific and often more pronounced for negative words (e.g., Scott et al., 2009; Kissler and Herbert, 2013; Yao et al., 2016; Schindler et al., 2019b), but have also been reported selectively for positive, e.g., happiness-related words (Briesemeister et al., 2014).

The visual N1 has been suggested as a first neural marker of context effects in word processing (Sereno and Rayner, 2003). Sereno et al. (1998) demonstrated the N1 to be sensitive to word-frequency effects in lexical decision. Moreover, this group also revealed that N1 amplitudes elicited by homonyms are modulated by meaning-biasing sentence context. For instance, N1 amplitude elicited by “bank” varied depending on whether the context contained “river” or “money” (Sereno et al., 2003). Scott et al. (2009) further showed an interaction of emotion with word frequency on the N1 in that the N1 was larger for high- than low-frequency negative words, whereas neutral words showed the opposite frequency modulation. Addressing neural effects of attributed social contexts, Schindler et al. (2019b) recently observed that valence effects on early brain potentials such as the N1 were elicited only when emotional trait adjectives were embedded in personally relevant communicative context—as a feedback personally targeted at the participant. When devoid of social embedding, the same emotional words elicited only late ERP amplifications (LPP). Together, the above findings highlight the role of the N1 as an early marker of context integration in word processing, in line with cascaded interactive processing models (see also Hauk et al., 2006). Whilst, at least in L1, the N1 has been shown to be sensitive to the emotional content of words in reading as well as to some semantic and social contexts, it is presently unknown whether it responds to mood contexts.

Some ERP studies have compared the processing of emotional words in L1 and L2. A common assumption in the bilingualism literature is that bilinguals are less sensitive to the emotional aspects in L2 (e.g., Pavlenko, 2012). Yet, extant electrophysiological studies point to similarities, especially in proficient bilinguals. For instance, Opitz and Degner (2012) testing late, but highly proficient bilinguals, report similar, if latency-shifted, results in both groups of bilinguals tested—German-French and French-German. Enhanced processing of emotional compared to neutral words was reflected in a larger EPN measured between 280 and 430 ms after word onset. While the EPN effect itself did not differ in amplitude between L1 and L2, it was delayed for L2. This suggests that emotional word content in L2 is processed in a less immediate way due to delayed lexical access. Similarly, in a lexical decision study with late German-Spanish and Spanish-German bilinguals, Conrad et al. (2011) reported morphologically highly similar ERPs across L1 and L2: Larger EPN and LPP for emotional words compared to neutral words in both languages. Again, particularly EPN latencies were delayed in L2. However, specifically the patterns for negative content in L2 differed between more and less proficient participants. While in the more proficient bilinguals they observed enhanced EPN and LPP for both positive and negative words in L2, in the less proficient ones, ERP modulations were restricted to positive words. This indicates that negative, but not positive emotional words may be treated in an unemotional manner in the L2, which is in line with a recent study showing that there is a learning effect for negative words in general, such that negative emotional words tend to be acquired later than positive words (Ponari et al., 2017).

Indeed, a growing body of studies reports flattened behavioral and/or electrophysiological responses particularly to negative word valence in participants’ L2. Several of these studies investigated the N400 ERP component (e.g., Wu and Thierry, 2012; Jończyk et al., 2016) which is a well-established marker of integration of words into their semantic context, particularly in sentence processing (e.g., Van Berkum et al., 1999).

Summing up, the second language research shows that, at least in proficient users, L2 should not be understood as totally unemotional, or driven by entirely different mechanisms than L1. Instead, the available evidence indicates weaker and delayed effects in L2 relative to L1, perhaps particularly regarding negative valence.

Recent interactional models of communication (e.g., Van Berkum, 2018, 2019) as well as embodiment theories (e.g., Matheson and Barsalou, 2018) emphasize that to gain insight into how individuals process and *experience* the affective content of words in communication, more attention should be paid to interactions between their respective *linguistic systems* and the accompanying *contexts*. One such context is mood, which has recently been conceptualized as an “overarching state of mind” with pervasive influence on all aspects of cognition (Herz et al., 2020). Arguably, in communicative interactions people draw on contextual information including their somatic states: how they feel when interacting (e.g., Zajonc, 1980; Higgins, 1998) to constrain cognition and guide their actions. Accordingly, recent models of affective language comprehension (e.g., Van Berkum, 2018, 2019) posit that in order to make sense of verbal content, people rely on their moods as sources of information (cf. Clore and Huntsinger, 2009). Still, studies on word processing in bilinguals have hardly explored to what extent mood-states modify the processing of semantic and affective word content.

A recent functional magnetic resonance imaging (fmri) study showed mood effects on language lateralization in general, revealing left lateralization of word fluency in anterior insula during happiness, and right hemisphere dominance during sadness (Costanzo et al., 2015). Bilingualism research has also found lateralization differences between L1 and L2 (e.g., D’Anselmo et al., 2013; Román et al., 2015), the combined findings suggesting that moods might differently impact neural processing of L1 and L2. So far, research targeting mood-effects in bilinguals boils down to one study testing mood and creativity (Kharkhurin and Altarriba, 2016), showing that positive mood enhanced creativity in participants’ dominant language, while negative mood boosted creativity in the non-dominant language.

Overall, it stands to reason that mood effects in either L1 or L2 should be most pronounced when emotional contents are processed, in line with the general ideas of mood-congruent processing (Bower, 1981) or affective priming (Klauer and Musch, 2003). Focusing on L1, several older behavioral studies have found mood-congruence effects for specific categories of words, but not for mood-valence agreement more broadly. That is, when happy or sad mood induction preceded lexical decisions on happiness- or sadness-related words, mood-congruent acceleration of reaction times was found (Niedenthal et al., 1994, 1997; Olafson and Ferraro, 2001; Ferraro et al., 2003), but the effect did not extend to positive or negative words in general (Niedenthal et al., 1994, 1997). Using a more extensive and more tightly controlled stimuli set than initial studies did, Sereno et al. (2015) recently observed faster reaction times in both a positive and a negative mood group compared to the control group (no mood induction). Moreover, whereas in positive mood reaction times were faster for both positive and negative words than for the neutral ones, in negative mood reaction times were fastest specifically for positive words, similar to what was found in the group without mood induction. This pattern was explained in terms of a general arousal-driven response acceleration in positive mood, in line with a motivated attention account that posits privileged processing of emotional content regardless of its valence (see also Kuperman, 2015). By contrast, automatic vigilance (Pratto and John, 1991) was suggested to operate in negative mood. Automatic vigilance refers to more pronounced attention capture, and delayed attentional disengagement from negative stimuli, therefore yielding faster reaction times for positive relative to negative words.

Kiefer et al. (2007) studied ERP correlates of mood effects on encoding of positive and negative adjectives. They specifically hypothesized that good, but not bad mood would facilitate mood-congruent processing (Fiedler, 2001). Empirically, they observed valence differentiation only in good mood, but not necessarily always reflecting a mood-congruent pattern: Early (200–350 ms) valence-dependent ERP differences over left central scalp regions occurred only in good mood, with negative words eliciting more negativity than positive words, reflecting mood incongruence. Between 350 and 500 ms, also in good mood only, an N400-like ERP was less negative-going for positive than for negative words, suggesting facilitated processing of positive words in good mood. In the LPP-window (500–650 ms), again, valence modulated ERPs only in good mood: Negative words elicited a more positive potential than positive words, specifically over frontal sites and originating in frontal and temporal regions. Thus, valence differentiation and recruitment of language-related brain regions were stronger for good relative to bad mood, but not necessarily in a consistent mood-congruent manner. Herring et al. (2011), investigating ERP correlates of affective word priming also found slower reaction times to affectively incongruent than congruent targets, and a larger LPP to these affectively incongruent targets, whereas N400 was insensitive to evaluative prime-target congruency in that study.

Prior research has indicated that words with emotional meaning need not always evoke representations of emotional content/feelings (e.g., Niedenthal et al., 1994). It might therefore be instrumental to use a task that taps directly into emotional aspects of word meanings. Therefore, similar to Herring et al. (2011), we employed an *evaluative decision task*, which directs participants’ attention to the emotional representation of the word meaning. Unlike tasks that call for lexical access solely, an emotion evaluation task should direct participants’ attention to the emotional content, thereby potentially also enhancing somatic representations of words’ meanings, which might amplify brain responses to words’ emotional content and even facilitate mood congruence across broad valence categories.

In sum, here, we investigate whether and how happy and sad moods will impact evaluative word processing in bilinguals. Given previous evidence from lexical decisions (Sereno et al., 2015), we expect faster responses and larger amplitudes for both positive and negative-neutral words in happy mood in L1. This pattern would be in line with predictions based on motivated attention (Kuperman, 2015). In sad mood, automatic vigilance may operate, which should be reflected in delayed responses to negative words (see also Sereno et al., 2015). On the neurophysiological level, stronger valence differentiation is expected in happy relative to sad mood (Kiefer et al., 2007). We analyze N1, EPN, N400, and LPP brain potentials regarding their modulations by mood and valence in L1 compared with L2. The full sequence of ERPs is assessed to determine the theoretically important temporal stages of potential interactions between mood, language, and word valence. Previous research has pointed to N1 as the first locus of integration between content and context, suggesting it as the first time-window of interactions between mood, word valence, and language status. EPN has consistently shown higher amplitudes to emotional than to neutral words, with its peak delayed in L2. N400 is a general marker of semantic integration whose amplitude is commonly larger for L2 (e.g., Ardal et al., 1990). N400 has also been found to be sensitive to emotional content (Herbert et al., 2008; De Pascalis et al., 2009; Holt et al., 2009; Moreno and Vázquez, 2011), its emotion modulation sometimes differing between L1 and L2 (Wu and Thierry, 2012; Martin et al., 2013; Jończyk et al., 2016). Mood-specific valence effects have also been reported on the N400 (Kiefer et al., 2007). Therefore, N400 could be another locus of integration of mood context with emotional content, which could further differ between L1 and L2. Finally, the LPP has been shown to be emotion-sensitive, including sensitivity to evaluative incongruence in priming (Herring et al., 2011) and mood sensitivity (Kiefer et al., 2007), but any differences between L1 and L2 remain to be explored. In order to specifically compare arousal- and valence-driven effects on the aforementioned components, we follow-up on any significant interactions with emotional content with pairs of linear and quadratic contrast. This allows us to distinguish between u-shaped (quadratic) effects that apply to both positive and negative content and are indicative of arousal-driven motivated attention effects, and valence-specific linear contrasts that differentiate between positive and negative contents, in line with predictions of automatic vigilance models. This strategy is commonly used in the emotion literature (e.g., Lang et al., 1993; Schindler et al., 2019a).

## Materials and Methods

### Participants

Twenty-seven student participants were recruited at Bielefeld University. They provided written informed consent according to the Declaration of Helsinki and participated either in partial fulfillment of a course requirement or were independently recruited via flyers and received 20 Euros for taking part in an experiment consisting of two experimental sessions on separate days. Of the 27 participants four had to be excluded. Two did not return for the second experimental session, one of the course participants was not a German native speaker and one participant indicated a current attention deficit hyperactivity disorder (ADHD) diagnosis on medical history screening and had markedly increased depression scores on the Beck Depression Inventory (BDI, Beck et al., 2001). Thus, data from 23 participants were included in the analysis. All participants spoke German as their (L1). They reported using both German and English on an everyday basis, in both formal and informal contexts, yet with L1 being their dominant language (see Tables 1, 2). Our participants were late learners of English as their L2, which they learnt in formal school settings in Germany. Their proficiency level in English was assessed via an on-line LexTALE test (Lemhöfer and Broersma, 2012), whose mean result indicated B2 proficiency level according to the Common European Framework of Reference for Languages (B2 cut-off > 60). In line with De Groot (2011), demographic information, and proficiency ratings, our participants are classified as upper intermediate/advanced, unbalanced, late English-German bilinguals. Due to experimenter error, LexTale scores are missing for two participants. All included participants were right-handed and free from acute psychiatric or neurological disorder as indicated by self-report. None of the included participants exhibited elevated anxiety and depressions scores as reflected on the State-Trait Anxiety Inventory (STAI, Spielberger et al., 1999) and the BDI. For full demographic information (see Table 1). Self-reported language history and communication skills according to the Language History Questionnaire (Li et al., 2014) are detailed in Table 2.

**TABLE 1:** Demographic information for the participants.

Variable	(*N* = 23)
Gender female/male	18/5
Age	24.9 (19–39, 4.3)
BDI Score	5 (0–12, 3.9)
STAI trait	35.61 (24–51, 8.3)
STAI state session 1	32.7 (23–46, 5.75)
STAI state session 2	31.8 (24–40, 4.9)
LexTale score	69.5 (9.15, 48–87)

*BDI, Beck Depression Inventory; STAI, State-Trait Anxiety Inventory. LexTale: Language Proficiency.*

**TABLE 2:** Linguistic information for the participants (Language History Questionnaire—LHQ; Li et al., 2014): self-reported information on the Age of acquisition of communicative competencies in English; Self-reported proficiency (1–7 scale), and daily time (in hours) spent using English.

Age of acquisition of English		Self-reported proficiency in English		Daily use of English (hours/day)	
Speaking	9.33 (1.77)	Speaking	5.19 (0.93)	Watching TV	0.8 (1.0)
Reading	9.52 (1.47)	Reading	5.57 (0.84)	Reading for fun	0.5 (0.5)
Writing	9.76 (1.27)	Writing	5.43 (0.87)	Interacting via the Internet	0.7 (0.6)
		Listening	5.38 (0.86)	Speaking with friends	0.5 (0.7)

### Stimuli

#### Word Material

Thirty six positive, negative, and neutral German adjectives were selected from the Berlin Affective Word List—Reloaded (BAWL-R, Võ et al., 2009). Adjectives appropriately differed in valence and arousal and were matched regarding concreteness, word length, word frequency, orthographic neighborhood density, and bigram frequency (see Table 3).

**TABLE 3:** Means for the set of German word attributes are given with standard deviations in parentheses; valence and arousal values are derived from the Berlin Affective Word List revised (BAWL-R; Võ et al., 2009) BAWL-R values range from −3.5 to + 3.5; lexicographic values come from dlex (Heister et al., 2011); means sharing the same superscript do not differ statistically.

Adjectives	Positive	Neutral	Negative
Valence	2.0 (0.06)^a^	0.04 (0.07)^b^	−2.0 (0.04)^c^
Arousal	2.9 (0.07)^a^	2.3 (0.08)^b^	3.1 (0.11)^a^
Concreteness	3.2 (0.14)^a^	3.1 (0.18)^a^	3.4 (0.15)^a^
Word length	6.6 (0.21)^a^	6.9 (0.19)^a^	7.1 (0.16)^a^
Word frequency (dLex)	31.4 (13.74)^a^	21.8 (7.17)^a^	20.0 (9.19)^a^
Orthographic neighborhood	0.7 (0.22)^a^	0.8 (0.18)^a^	0.6 (0.20)^a^
Bigram frequency	167086.6 (20480.01)^a^	175062.9 (19243.04)^a^	181801.1 (22722.29)^a^

*Means not sharing superscripts differ. Comparisons are based on Fisher’s LSD test post-hoc comparisons.*

To create a corresponding English stimulus set, these words were translated into English. Corresponding values for English are given in Table 4.

**TABLE 4:** Means for the word attributes for the set of English words.

Adjectives	Positive	Neutral	Negative
Valence	6.9 (0.7)^a^	5.4 (0.9)^b^	3.1 (0.8)^b^
Arousal	4.8 (0.9)^a^	3.6 (0.6)^b^	4.6 (1.0)^a^
Concreteness	2.3 (0.5)^a^	2.5 (0.8)^a^	2.4 (0.5)^a^
Word length	6.8 (1.7)^a^	6.8 (1.8)^a^	6.7 (1.9)^a^
Word frequency (zipf)	3.9 (0.6)^a^	3.4 (0.8)^a^	3.6 (0.9)^a^
Orthographic neighborhood	6.8 (1.7)^a^	3.7 (4.1)^a1^	6.4 (9.0)^a^

*Standard deviations in parentheses; means sharing the same superscript do not differ statistically. Means not sharing superscripts differ. Evaluations of the English word set for valence, arousal come from Warriner et al. (2013); scales range 1–9; zipf frequencies from SUBTLEX_US (van Heuven et al., 2014); concreteness values from Brysbaert et al. (2014); scales range: 1–5 (abstract-concrete); Orthographic Neighborhood based on CLEARPOND (Marian et al., 2012). ^1^Unfortunately a number of neutral words was missing from the CLEARPOND set, hence the larger standard deviation relative to positive and negative word valence.*

### Mood Induction

For mood induction, six different short movie excerpts with an average duration of 60 s were used. Three of these were happy and three were sad. The excerpts had been previously validated to generate the expected significantly different happy and sad moods states.

The happy clips were: “The Lion King: Final Scene (01:22:36–01:23:23; 47 s),” “The Lottery Ticket: Winning the Lottery” (00:23:53–00:25:07; 01:14 min), and “An Officer and Gentleman: Carried Away” (001:55:42–01:56:53; 01:11 min). The sad clips were: “The Lion King: Mufasa’s Death” (00:36:37–00:37:48; 01:11 min), “The Green Mile: John Coffee’s Death” (02:47:55–02:49:11; 01:16 min), and “The Champ: Final Scene” (01:53:08–01:54:05; 00:57 min). According to Gross and Levenson (1995), the final scene from “The Champ” is the most effective clip for inducing sad mood in their set. The clips were taken from the German and English versions of the movies, respectively.

### Procedure

The experiment was divided into two sessions, taking place on two separate days. On the first day, upon arrival at the laboratory, participants were introduced to the EEG set-up and the aim of the study was explained to them in general terms as a study on emotion in language processing in their L1 and L2. While the electrodes were being attached, participants completed several questionnaires: On the first appointment, a demographic and health questionnaire, the BDI, and the STAI state questionnaire were administered. On the second appointment the STAI state and trait questionnaires as well as the LHQ and the LexTale test were given.

After electrode placement, the study was explained in more detail: Participants were told that they would see short video clips that they should watch attentively. Thereafter, they would be presented with words that they should categorize via button-press (left arrow, up-arrow or right arrow) as positive, negative, or neutral. This procedure would repeat several times after which the words would switch to a different language in a separate language block (English or German, respectively).

Words were presented in three blocks, each preceded by a short movie clip. The valence of the mood induction remained constant for three blocks in a row. Word blocks consisted of 36 items each, 12 positive, 12 negative, and 12 neutral. Word order was randomized within each block separately. Words were presented in white font (Arial, 40 pts) on a black screen, each for 616 ms, followed by a white fixation cross prompting participants to respond. The fixation cross was presented for a randomly varying inter-stimulus interval (ISI) of 1.9–2.3 s.

After block 1 and 2, participants were given a short self-paced break to allow them to relax briefly. After the third block of each session, participants were asked to assess their emotional state, i.e., rating subjectively felt valence and arousal on a nine-point Self-Assessment Manikin scale (Bradley and Lang, 1994) as well as their level of current happiness or sadness, on a seven point Likert scale.

Then, participants were allowed a longer break and the experimental language and mood induction were switched. The above described procedure was repeated with mood inductions and words presented in the other language. The experiment was controlled via Presentation software^1^.

At the end of the first experimental session, another appointment was made for a second, analogously structured, experimental session. Experimental conditions were counterbalanced with the restriction that participants always underwent two different mood blocks and two different languages per session.

#### Analyses of Behavioral Data

Behavioral data were analyzed according to their match with predefined word categories. Number of word assignments per category (positive, neutral, and negative) as well as reaction times were analyzed within a response window of 1,500 ms following stimulus onset. Reaction times were corrected for outliers, excluding responses that exceeded ± 2 SD of the individual mean and recalculating the reaction time. Statistical analyses were performed using repeated measures ANOVA with the factors Mood (Happy, Sad), Language (L1: German, L2: English), and Word Content (positive, neutral, negative).

#### EEG Recording and Analyses

EEG was recorded from 32 BioSemi active electrodes^2^ sampled at 1,024 Hz. Two separate electrodes were used as ground electrodes, a Common Mode Sense active electrode (CMS) and a Driven Right Leg passive electrode (DLR), which formed a feedback loop that enabled measuring the average potential close to the reference in the A/D-box^3^. Four additional electrodes (EOG) placed near the outer canthi and below the eyes measured horizontal and vertical eye movement.

Pre-processing and statistical analyses were performed using BESA^4^ and EMEGS (Peyk et al., 2011). Offline, data was re-referenced to an average reference and a forward 0.16 Hz high-pass and a zero-phase 30 Hz low-pass filter were applied. Filtered data were segmented from 100 ms before word onset until 1,000 ms after stimulus presentation. The 100 ms before stimulus onset were used for baseline correction. Eye-movements were corrected using the automatic eye-artifact correction method implemented in BESA (Ille et al., 2002). ERP data were statistically analyzed with EMEGS (Peyk et al., 2011).

ERPs were averaged according to predefined word categories matched for other lexical variables (see section “Materials and Methods”) and analyzed in 5 different time windows and components, namely the N1 (125–200 ms), EPN1 (200–300 ms), EPN2 (300–400 ms), N400 (350–450 ms), and LPP (500–700 ms). Time-windows largely correspond to those in previous studies (see e.g., Herbert et al., 2008; Scott et al., 2009). We divided EPN into two time windows in order to be able to assess any processing delay for emotional content in L2 as suggested by previous research (Conrad et al., 2011; Opitz and Degner, 2012). Analyses were performed at two symmetrical occipital (O1, PO3, P3, P7 and O2, PO4, P4, P8) and temporal (CP5, T7, FC5, F7 and CP6, T8, FC6, F8) electrode groups for N1, EPN1, and EPN2 components. For N400 a fronto-central group consisting of Cz, Fz, FC1, and FC2 and for LPP a centro-parietal group comprising P3, CP1, Pz, P4, and CP2 were employed. Number and location of the grouped electrodes largely corresponded to the one presented by Scott et al. (2009) who also used four electrodes per cluster. As in Dehaene (1995) and according to the observed scalp topographies, we analyzed early negativities (N1 and EPN) at temporal as well as occipital sites.

Statistical analyses were conducted in EMEGS and SPSS 25. Analyses of variance (ANOVAs) were performed with the repeated measurement factors Mood Induction (happy, sad), Language (L1: German, L2: English), Word Valence (positive, neutral, negative) for behavioral data and N400 and LPP components. For the N1 and EPN components, laterality of Channel Group (left, right) was added to assess expected hemispheric asymmetries in language and mood processing. Significant higher-level ANOVAs were broken down into follow-up ANOVAs and the shapes of any valence-dependent (positive, neutral, negative) differences were determined with pairs of polynomial trend tests, comparing linear and quadratic trends, significant linear trends indicating valence-dependent effects and significant quadratic trends indicating arousal-driven effects (see also Lang et al., 1993; Schindler et al., 2019a). If the sphericity assumption was violated, degrees of freedom and *p*-values were corrected according to the Huynh-Feldt procedure. In line with the literature, we report uncorrected degrees of freedom and corrected *p*-values for better readability. Partial eta-squared (η_p_^2^) was estimated to describe effect sizes (Cohen, 1988).

## Results

### Behavior

#### Manipulation Check

Participants rated their moods as significantly more positive [*F*(1, 22) = 30.43, *p* < 0.001, η_p_^2^ = 0.58] after the happy than after the sad mood induction. Mood valence did not differ between L1 and L2 [*F*(1, 22) = 0.4, *p* = 0.53, η_p_^2^ = 0.02] and the effect of mood induction did not interact with language [*F*(1, 22) = 1.3, *p* = 0.27, η_p_^2^ = 0.06]. By contrast, mood induction did not impact self-rated arousal [*F*(1, 22) = 0.0, *p* = 1, η_p_^2^ = 0.0] in either language [*F*(1, 22) = 0.27, *p* = 0.61, η_p_^2^ = 0.01] and the interaction was likewise insignificant [*F*(1, 22) = 0.24, *p* = 0.63, η_p_^2^ = 0.01]. Self-rated sadness was higher following sad than happy mood induction [*F*(1, 21) = 27.34, *p* < 0.001, η_p_^2^ = 0.57], with no difference between the languages [*F*(1, 21) = 2.25, *p* = 0.15, η_p_^2^ = 0.1] and no interaction [*F*(1, 21) = 0.96, *p* = 0.34, η_p_^2^ = 0.04]. One participant failed to complete the sadness rating.

#### Word Evaluations

**FIGURE 1:**
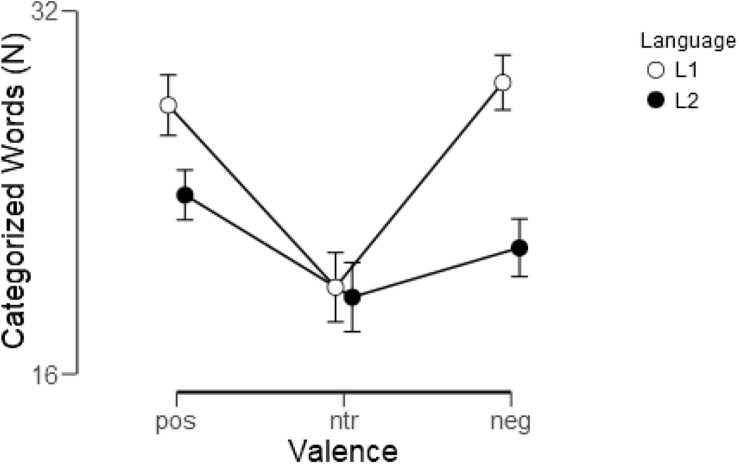
Assignment of words to the positive (pos), neutral (ntr), and negative (neg) valence categories in L1 (German) and L2 (English). Shown are means and standard errors.

An analysis of evaluations according to predefined valence categories revealed that in L1 considerably more words were evaluated as expected than in L2 [*F*(1, 22) = 48.74, *p* < 0.001, η_p_^2^ = 0.69]. Overall, more words were evaluated as either positive or negative than as neutral [*F*(2, 44) = 15.87, *p* < 0.001, η_p_^2^ = 0.42; quadratic: *F*(1, 22) = 19.08, *p* < 0.001, η_p_^2^ = 0.46, linear: *F*(1, 22) = 0.84, *p* = 0.37, η_p_^2^ = 0.04], but a highly significant interaction of valence and language [*F*(2, 44) = 21.0, *p* < 0.001, η_p_^2^ = 0.49] reflected that this was considerably more pronounced in L1 [quadratic: *F*(1, 22) = 42.24, *p* < 0.001, η_p_^2^ = 0.65; linear: *F*(1, 22) = 1.36, *p* < 0.25, η_p_^2^ = 0.06] than in L2 [quadratic: *F*(1, 22) = 4.64, *p* < 0.05, η_p_^2^ = 0.17], linear: *F*(1, 22) = 6.21, *p* < 0.05, η_p_^2^ = 0.22]. In particular, whereas in L1 considerably more words were assigned to both the positive [*t*(22) = 5.3, *p* < 0.001] and the negative [*t*(22) = 7.32, *p* < 0.001] than to the neutral category, in L2 assignment to positive differed from neutral [*t*(22) = 2.58, *p* < 0.05] whereas negative and neutral did not differ [*t*(22) = 1.48, *p* = 0.15]. Figure 1 shows how word evaluations were distributed across the valence categories in the two languages.

#### Reaction Times

**FIGURE 2:**
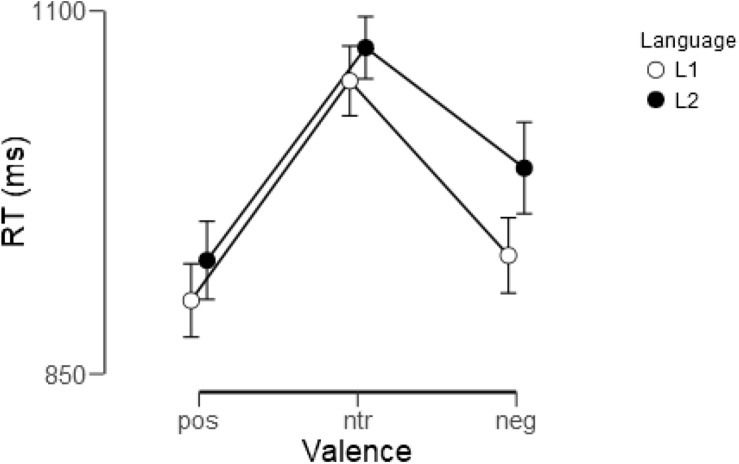
Reaction times (RT) for evaluation of positive (pos), neutral (ntr), and negative (neg) words in L1 (German) and L2 (English). Shown are means and standard errors.

As shown in Figure 2, words were evaluated faster in L1 (German) than in L2 (English) [*F*(1, 22) = 8.07, *p* < 0.01, η_p_^2^ = 0.27] and emotional words were evaluated faster than neutral ones [*F*(2, 44) = 41.1, *p* < 0.001, η_p_^2^ = 0.65]. A trend-level interaction indicated that participants took a little longer when they evaluated negative L2 words than negative L1 words [*F*(2, 44) = 2.74, *p* < 0.1, η_p_^2^ = 0.11], reaction times for the other two categories not differing. Reaction times for the individual experimental conditions are detailed in Table 5.

**TABLE 5:** Reaction times for evaluative decisions.

Mood	Language	Valence	Mean	*SD*
Happy	L1	pos	884.89	123.52
		ntr	1046.67	126.22
		neg	913.79	129.39
	L2	pos	926.34	119.39
		ntr	1072.42	148.30
		neg	989.23	142.21
Sad	L1	pos	916.39	125.72
		ntr	1056.96	123.47
		neg	949.56	113.18
	L2	pos	930.12	126.15
		ntr	1076.91	111.74
		neg	994.41	152.74

#### ERP data

##### Occipital N1

Left-lateralization of the occipital N1 in word processing was reflected in a main effect of channel group [*F*(1, 22) = 5.9, *p* < 0.05, η_p_^2^ = 0.21].

**FIGURE 3:**
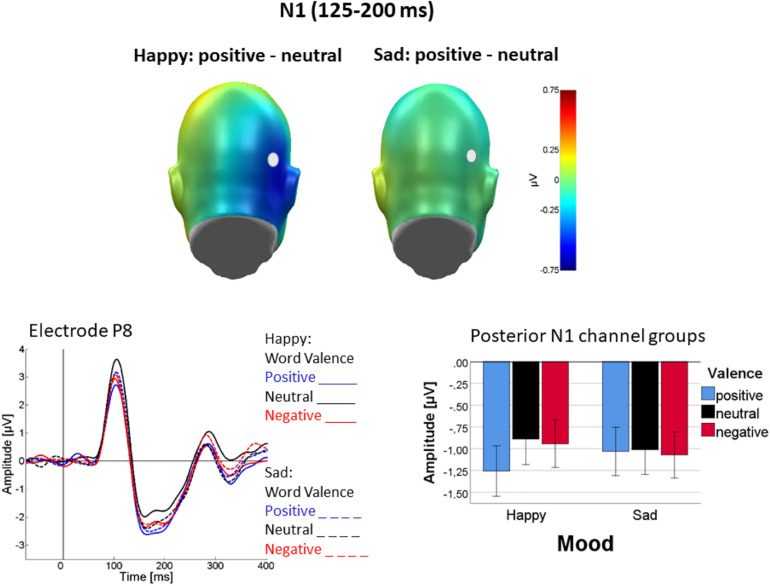
Difference topographies illustrating mean N1 activation (125–200 ms) during processing of emotional (positive and negative) minus neutral adjectives in happy (top left) and sad (top right) mood. Representative sensor P8 illustrates the ERP time course for the different conditions (solid: happy mood; dashed: sad mood; word valence: blue—positive, black—neutral, red—negative. Bar plots show posterior N1 activity averaged across both occipital sensor groups and the entire N1 interval (125–200 ms). Error bars are standard errors.

Moreover, as shown in Figure 3, valence differentiation was found following happy but nod sad mood induction, as evident in an interaction of Mood with Valence [*F*(2, 44) = 4.62; *p* = 0.02; η_p_^2^ = 0.17]. In detail, in happy mood an effect of valence was found [*F*(2, 44) = 9.65; *p* < 0.01, η_p_^2^ = 0.30] in that N1 was largest for positive words, negative and neutral not differing [linear: *F*(1, 22) = 12.87, *p* < 0.01, η_p_^2^ = 0.37; quadratic: *F*(1, 22) = 6.82, *p* < 0.05, η_p_^2^ = 0.24]. By contrast, no valence effect was seen following sad mood induction [*F*(2, 44) = 0.25, *p* = 0.8, η_p_^2^ = 0.01]. Figure 3 illustrates the interaction of mood and valence, showing the valence effect in happy but not in sad mood. Figure 3 also suggests valence differentiation in the occipital N1 to be primarily driven by the right hemisphere, but the interaction was not significant [Valence × Channel Group, *F*(2, 44) = 2.92, *p* = 0.06; η_p_^2^ = 0.12, see also Figure 3]. Finally, a three-way interaction of language with mood and channel group was found [*F*(1, 22) = 5.54, *p* < 0.05, η_p_^2^ = 0.2] in that mood affected N1 lateralization differently in the two languages. This effect was mainly driven by temporal rather than occipital activity (see detailed analysis below). No other main effects or interactions were significant.

#### Lateral N1

**FIGURE 4:**
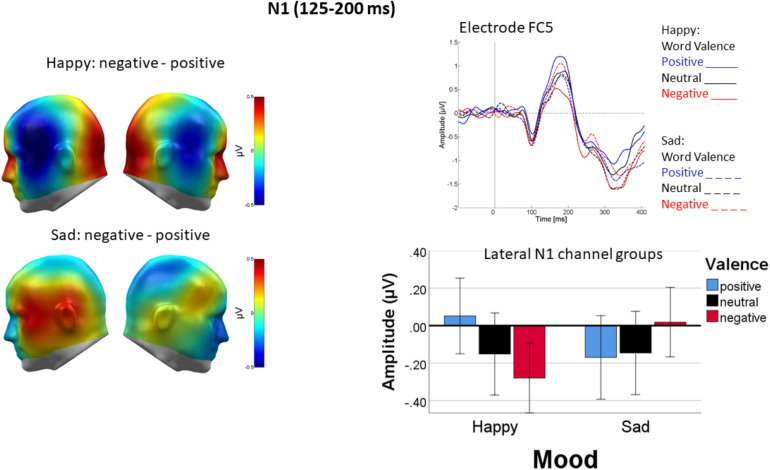
Differential processing of negative versus positive words over temporal cortices following happy but not sad mood induction. **Left panel**: Difference topographies of average activity in the N1 time window (125–200 ms). **Right panel**: Representative sensor FC5 (top) and bar plot showing mean activity averaged across both temporal sensor groups. Error bars are standard errors.

Over lateral parts of the N1, mood induction interacted with valence [*F*(2, 44) = 6.96, *p* < 0.01, η_p_^2^ = 0.22, see Figure 4]. Following happy mood induction, the valence effect [*F*(2, 44) = 5.66, *p* < 0.01, η_p_^2^ = 0.21] occurred because ERPs elicited by negative words were more negative-going than ERPs elicited by positive words, neutral words falling in between [linear: *F*(1, 22) = 15.1, *p* < 0.01, η_p_^2^ = 0.4; quadratic: *F*(1, 22) = 0.15, *p* = 0.7, η_p_^2^ = 0.007]. This was not the case following sad mood induction [*F*(2, 44) = 1.64, *p* > 0.1, η_p_^2^ = 0.07].

**FIGURE 5:**
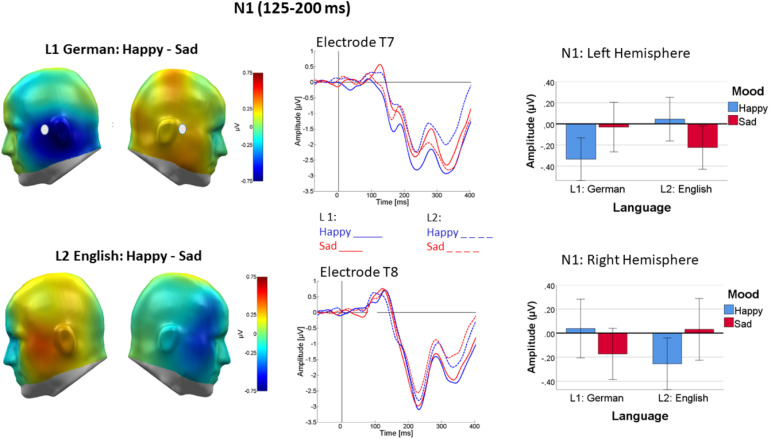
Difference topography of word processing following happy minus sad mood induction in L1 **(top left)** and L2 **(bottom left)** in the N1 time window (125–200 ms). **Middle panel**: ERP at representative sensors T7 **(top row)** and T8 **(bottom row)**, revealing stronger mood-dependent lateralization in happy mood in L1 than L2. Right panel shows the pattern as bar charts separately for the left and right temporal sensor groups and L1 (German) on the left and L2 (English). Error bars are standard errors.

As a main finding in this time-window, mood induction impacted the lateralization of word processing differently in L1 and L2 [Mood × Language × Channel Group: *F*(2, 44) = 11.045, *p* < 0.005, η_p_^2^ = 0.33]. As shown in Figure 5, in L1 (German), mood induction had a highly significant effect on the lateralization of word processing [*F*(1, 22) = 11.35, *p* < 0.005, η_p_^2^ = 0.34]. N1 was more negative over the left than over the right channel group following happy mood induction [*F*(1, 22) = 4.8, *p* < 0.05, η_p_^2^ = 0.18] with no lateralization following sad mood induction [*F*(1, 22) = 1.05, *p* < 0.32, η_p_^2^ = 0.01]. An interaction of mood and channel group was also present in L2 [*F*(1, 22) = 6.67, *p* < 0.05, η_p_^2^ = 0.230.23] and its pattern seemed reversed (see Figure 5, bottom row). However, in L2 follow-up tests were not significant.

There was also a three-way interaction of language with valence and channel group [*F*(2, 44) = 3.352, *p* = 0.044, η_p_^2^ = 0.13], but follow up ANOVAs were all insignificant.

Furthermore, a complex four-way interaction of mood with language, valence, and channel group [*F*(2, 44) = 4.722, *p* = 0.014; η_p_^2^ = 0.17] occurred. However, none of the follow-up tests was significant.

##### Occipital EPN1

**FIGURE 6:**
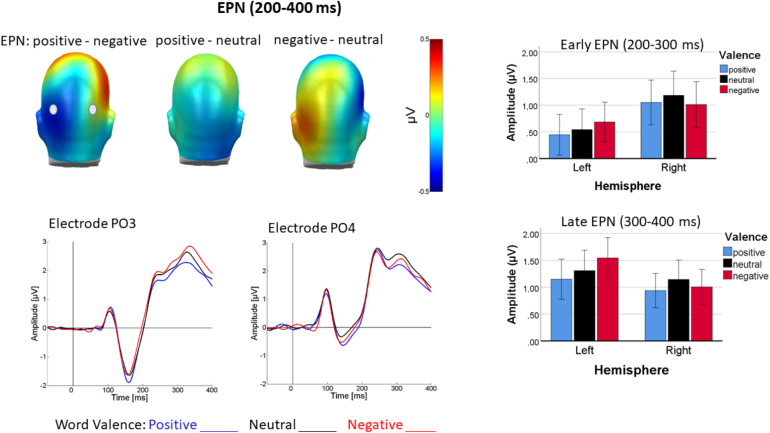
Top left shows difference topographies for positive minus negative, positive minus neutral and negative minus neutral words averaged across a time window of 200–400 ms (EPN1 and EPN2). Bottom left: ERP time course for positive (blue), neutral (black), and negative (red) words at representative sensors PO3 **(left)** and PO4 **(right)**. Right panel: Bar charts of average activity in left and right occipital sensor groups elicited by the different word valences from 200 to 300 ms (top) and 300 to 400 ms (bottom). Error bars are standard errors.

In the early occipital part of the EPN, valence interacted with channel group [*F*(2, 44) = 3.57, *p* < 0.05, η_p_^2^ = 0.14] reflecting linear valence discrimination, with more negative-going ERPs for positive than negative words over the left occipital cortex [linear: *F*(1, 22) = 5.67, *p* < 0.05, η_p_^2^ = 0.20; quadratic: *F*(1, 22) = 0.1, *p* = 0.76, η_p_^2^ = 0.76], whereas over right occipital cortex valence discrimination was insignificant [*F*(1, 44) = 1.11, *p* > 0.05, η_p_^2^ = 0.05]. Figure 6 illustrates this pattern.

##### Lateral EPN1

Over lateral sensors, an interaction of mood with valence occurred [*F*(2, 44) = 5.33, *p* < 0.01, η_p_^2^ = 0.19]. Its pattern was descriptively similar to the N1 (see Figure 4), but follow-up test were not significant (*p*_s_ > 0.07). An interaction of valence and channel group was also found [*F*(2, 44) = 4.096, *p* = 0.023, η_p_^2^ = 0.16], but follow-up tests could not resolve it (all *p*s > 0.1). No other effects approached significance (*p* > 0.11).

##### Occipital EPN2

For the later part of the EPN, the interaction of valence with channel group persisted [*F*(2, 44) = 3.51, *p* < 0.05, η_p_^2^ = 0.14]. The valence effect over the left channel group [*F*(2, 44) = 4.54, *p* < 0.05, η_p_^2^ = 0.17] was due to linearly more negative-going potentials for positive than negative words [linear: *F*(1, 22) = 13.42, *p* < 0.36, η_p_^2^ = 0.38; quadratic: *F*(1, 22) = 0.084, *p* = 0.77, η_p_^2^ = 0.004], whereas the descriptively inversely u-shaped valence differentiation over the right hemisphere was insignificant [*F*(2, 44) = 1.03, *p* > 0.1, η_p_^2^ = 0.05]. Figure 6 illustrates EPN modulation by emotional words for both analyzed time-windows. No other effects were significant (*p* > 0.07).

##### Lateral EPN2

Over lateral temporal sensors, an effect of language occurred [*F*(1, 22) = 4.34, *P* < 0.05] in that ERPs were more negative-going for L1 (German) than L2 (English). An interaction of mood and word valence [*F*(2, 44) = 6.57, *p* < 0.01, η_p_^2^ = 0.23] resembled the pattern found for the N1 and can be seen in the sensor tracings in Figure 4. It was due to a valence effect following happy mood induction [*F*(2, 44) = 4.89, *p* < 0.01, η_p_^2^ = 0.18] such that negative words were most negative going [linear: *F*(2, 22) = 6.57, *p* < 0.05, η_p_^2^ = 0.23; quadratic: *F*(2, 44) = 3.26, *p* = 0.08, η_p_^2^ = 0.13], whereas the valence effect in sad mood was not significant [*F*(2, 44) = 2.5, *p* = 0.09, η_p_^2^ = 0.1]. In particular, negativity elicited by negative words was more pronounced following happy than following sad mood induction [*t*(22) = −2.79, *p* < 0.05]. An interaction of valence with channel group [*F*(2, 44) = 3.361, *p* < 0.05, η_p_^2^ = 0.13] was also present, but none of the follow-up tests was significant.

##### N400

On the N400, a main effect of language [*F*(1, 22) = 5.061; *p* = 0.035, η_p_^2^ = 0.19] emerged, reflecting a larger N400 in L2. No other main effects or interactions occurred (*p*s > 0.2).

#### LPP

**FIGURE 7:**
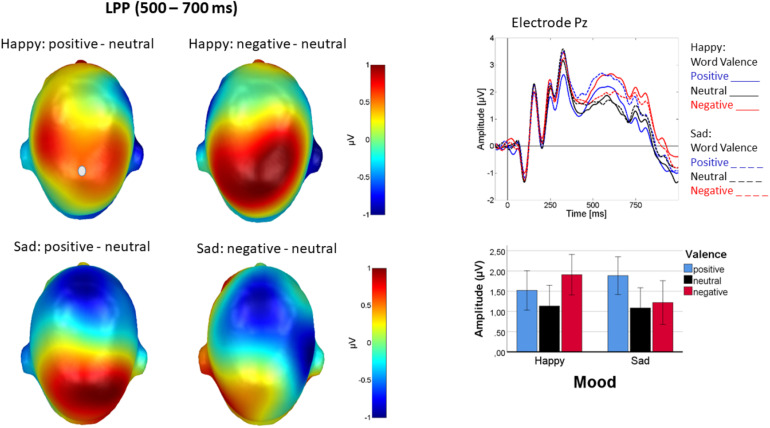
LPP difference topographies depicting word processing in happy mood **(left panel, top row)** and sad mood **(left panel, bottom row)**. Top of right panel shows ERPs at sensor Pz, where solid lines represent happy mood and dashed lined sad mood. Blue is positive, red negative, and black neutral word valence. A bar chart **(bottom right)** depicting mean activity from the centro-parietal sensor group across the LPP time interval (500–700 ms) illustrates the interaction. Particularly mood-incongruent words elicited higher LPP amplitudes. Error bars are standard errors.

On the LPP, a main effect of word valence [*F*(2, 44) = 5.925, *p* = 0.005, η_p_^2^ = 0.21], reflecting higher LPP amplitudes for both positive and negative rather than for neutral words [linear: *F*(1, 22) = 0.63, *p* < 0.43, η_p_^2^ = 0.03], quadratic: [*F*(1, 22) = 10.61, *p* <0.005, η_p_^2^ = 0.47], and an interaction of mood and word valence [*F*(2, 44) = 8.815; *p* = 0.001, η_p_^2^ = 0.29] were found. Figure 7 illustrates that the interaction reflected cortical accentuation of mood-incongruent content. The valence effect following positive mood induction [*F*(2, 44) = 9.37, *p* < 0.001, η_p_^2^ = 0.30] arose, because here negative words elicited highest amplitudes and amplitudes for positive words fell between negative and neutral [linear: *F*(1, 22) = 5.46, *p* < 0.05, η_p_^2^ = 0.20], quadratic: [*F*(1, 22) = 12.35, *p* < 0.005, η_p_^2^ = 0.36]. By contrast, for the valence effect following negative mood induction [*F*(2, 44) = 5.65, *p* < 0.01, η_p_^2^ = 0.20], positive words elicited highest amplitudes, with little difference between negative and neutral words [linear: *F*(1, 22) = 7.23, *p* < 0.05, η_p_^2^ = 0.25], quadratic: [*F*(1, 22) = 4.24, *p* = 0.05, η_p_^2^ = 0.16]. No other effects approached significance (*p* > 0.13).

## Discussion

In the present study, we investigated how the processing of emotional word content is modulated by moods. We specifically compared how happy and sad moods affect the processing timeline of emotional adjectives when participants responded to them in L1 (German) and L2 (English), respectively. In two mood induction conditions, the same sets of positive, neutral and negative trait adjectives were presented either in participants’ L1 or L2 while they evaluated the emotional content of the presented adjectives. Based on previous literature, we considered predictions from mood-congruence (Bower, 1981; Niedenthal et al., 1994), motivated attention (Kuperman, 2015), automatic vigilance (Pratto and John, 1991) and more emotion differentiation in good mood (Fiedler, 2001; Kiefer et al., 2007). Against these backgrounds, we aimed to establish empirically the timeline of mood and word valence interactions, examining specifically whether these effects would be observed already at the early (N1, EPN) ERPs and whether the brain potentials evoked in L1 and L2 would differ in amplitude and latency.

**Behavioral data** showed that participants differed in their responses to emotional word content in their L1 and L2. Their evaluations were faster in L1, their functionally dominant language, than in L2. They were also faster on both positive and negative emotional words than on neutral words, which is theoretically in line with the pattern expected by the motivated attention model (see Kuperman, 2015). However, reaction times for negative L2 words, while being considerably faster than for neutral ones, were slower than for positive L2 words which provides further evidence for attenuated processing of negative contents in L2 as suggested by some previous research (Wu and Thierry, 2012; Sheikh and Titone, 2016; Baumeister et al., 2017; Jończyk et al., 2019). Also, in L1, relative to L2, considerably more words were evaluated according to predefined word valence. This finding, indicating greater emotional distance in L2 relative to emotional words in L1, is consistent with a range of studies exploring a phenomenon referred to as a *foreign language effect*, which posits that when functioning in L2, people adopt a more utilitarian thinking style, which leads to different decision patterns as compared to when they operate in their L1 (Keysar et al., 2012; Costa and Sebastián-Gallés, 2014; Hayakawa et al., 2017).

Evaluation and reaction time data showed no effect of mood induction in either language, although in both languages self-rated mood questionnaires showed an expected difference on subjective valence and sadness ratings. One reason for this might be that the evaluative decision task, while making the affective dimension more salient than lexical decision does, also results in longer and more variable reaction times. Moreover, unlike anticipated, explicit emotion evaluation may override any more subtle implicit processes that mood might have on overt behavior. At any rate, present behavioral data provide no evidence in favor of mood-congruent processing suggested by older lexical decision studies (Niedenthal and Setterlund, 1994; Niedenthal et al., 1997) or any other of the above models, although a more recent study (Sereno et al., 2015) revealed mood effects on lexical decisions. However, mood clearly impacted the neurophysiology of word processing.

### Neural Effects of Mood Induction

#### N1

Cortical differentiation of word valence for happy, but not for sad mood, already occurred on the N1. This was observed over occipital as well as temporal areas, although the pattern differed in that over occipital regions enhancement of N1 to positive words was mood-congruent, whereas over temporal areas mood-incongruent negative words induced more negativity. More pronounced valence differentiation in happy than sad mood, as seen in several time windows, is in agreement with findings by Kiefer et al. (2007) who hypothesized that specifically in positive moods, contents would be encoded in an assimilative manner, favoring mood-congruent processing (Fiedler, 2001). Whereas we found stronger valence differentiation in word-evoked ERPs in happy mood across all early components, the pattern was not always mood-congruent, which was previously observed by Kiefer et al. (2007).

Strikingly, regardless of word valence, mood induction affected the lateralization of word-evoked N1 over temporal sites, the pattern differing between the two languages. In L1, the N1 component was strongly left-lateralized following happy mood induction, which was not the case following sad mood induction. This pattern was absent in L2. In line with previous research, both the early interaction of mood and valence and the interaction of mood and language status confirm the N1 time-window as an important, and possibly the first, window of integration of word meaning with its presentation context (Sereno et al., 2003; Schindler et al., 2019a). Our data extend this notion from meaning-biasing sentence context (Sereno et al., 2003) and putative social contexts (Schindler et al., 2019a) to mood as an emotional context of word processing. Crucially, results demonstrate that lateralization of word processing is malleable by mood-induction and that these effects further differ between L1 and L2. This novel finding was valence-general and resonates with the results of a recent fmri study that likewise indicated that mood-states affect language lateralization, with stronger left-lateralization in insular cortex in positive mood (Costanzo et al., 2015). Given the topography of the lateral N1, we observed, the insula might well be one source of this effect. Costanzo et al. (2015) also showed that mood affected language lateralization differently in atypically lateralized people. Given the evidence that L2 often exhibits a different, more rightward lateralization than L1, particularly in related languages (D’Anselmo et al., 2013) as are German and English, the apparently inverted mood effect in L2 is in general agreement with Costanzo et al.’s findings. A differential pattern of right hemisphere (RH) activation in L2 would be consistent with the more widespread neural activation in the RH (e.g., Román et al., 2015; Połczyńska et al., 2017) found especially in the second language of less highly functional bilinguals (as is the case with our L1 dominant participants). Going beyond the language-general effects, additional higher-order interactions suggested that some of the early mood induction effects on neural correlates of word processing in L1 versus L2 may be valence specific, but since follow-up testing could not clearly identify their origin, further research with more participants will be needed to clarify this issue.

#### EPN

We divided the subsequent EPN in two time-windows, one from 200 to 300 ms and one from 300 to 400 ms to address the possibility of delayed valence processing in L2 (Conrad et al., 2011; Opitz and Degner, 2012). In line with ample previous research (for review see e.g., Citron, 2012), the time-window between 200 and 300 ms, presently scored as early EPN, was emotion sensitive, albeit not reflecting the more often observed u-shaped, arousal-driven pattern, but linear valence discrimination with more negativity elicited by positive than negative words, at least over left occipital areas. Over left hemisphere sites, mood also impacted word processing in a valence-specific manner. Cortical valence differentiation was primarily present after happy mood induction, again in line with the findings by Kiefer et al. (2007). In general, the observed early cortical processing of emotional words was valence-specific, differentiating between positive and negative, which would neither be expected by a motivated attention account (Kuperman, 2015), nor fully in line with automatic vigilance (Pratto and John, 1991), since the early visual attention-sensitive ERPs responded selectively to positive rather than negative words. Previous research on emotional word processing typically revealed arousal-driven ERP modulations (Fischler and Bradley, 2006), at least during free-viewing (e.g., Kissler et al., 2007) or lexical decision (Schacht and Sommer, 2009b). Present results suggest that explicit evaluation accentuates valence-specific perceptual processing, apparently particularly in positive mood as already suggested by Kiefer et al. (2007). Surprisingly, however, the pattern was reversed over temporal regions, and apparently generally in higher-level processing (see below). This theoretically unexpected finding was observed in several time-windows, lending it conceptual credibility. It may reflect the need for alerting by an unexpected input, similar to what is sometimes seen as processing interrupt in the startle literature (Herbert and Kissler, 2010; Blumenthal, 2015). Mood effects over perceptual brain areas, by contrast, exhibited a mood-congruence pattern with larger amplitudes for the mood-congruent words. The latter portion of the EPN, between 300 and 400 ms, conceptually replicated what was seen in the early EPN as well N1. We found no evidence for delayed valence processing in L2 which would have been evident in an interaction of word valence with language in either of the EPN windows, which might be due to the relatedness of the two languages used. Instead, between 300 and 400 after word onset, over temporal areas, ERPs were generally more negative-going in L1 than in L2, probably reflecting a polarity reversal of the fronto-central N400.

#### N400

On the N400, a main effect of language was prominent. In line with the ERP literature pointing to N400 as an index of more wide-spread search in language networks (e.g., Kutas and Hillyard, 1980; Kutas and Federmeier, 2000, 2011), we found more negative N400 amplitudes in L2 relative to L1. Larger N400 in L2 than in L1 has been previously observed in word and sentence processing tasks (e.g., Ardal et al., 1990; Moreno and Kutas, 2005; Martin et al., 2013). For instance, in visual processing of words and sentences, bigger N400 amplitudes for L2 stimuli typically have been interpreted as indices of cognitive effort increase, i.e., more extensive lexical search for the L2 word meaning, or more difficulty in integrating L2 word meaning with the representation of the ongoing context (e.g., Moreno and Kutas, 2005; Thierry and Wu, 2007; Martin et al., 2013). Therefore, more negative amplitudes evoked in L2 relative to L1, as we observed here, should indicate of more extensive lexical search in L2 irrespective of mood. German-English bilinguals were employing more cognitive resources to perform the evaluation task in English (L2) than in German (L1). This finding contributes to the body of literature already showing that the N400 amplifications might be qualitatively different in the two languages of bilingual individuals, with factors such as language proficiency, or age of L2 acquisition most likely modulating N400 amplitude. However, unlike shown in previous sentence level (e.g., Federmeier et al., 2001; Pinheiro et al., 2013) or word level (Kiefer et al., 2007) research, no mood effects were present on the N400. This might be due to a combination of word level processing and the evaluative decision task that might have shifted neural mood and content effects in time, perhaps pushing them into earlier negativity or later positivity windows. No effects of word valence were found on this component either, which across emotional word processing studies is not unusual as only some studies report emotion effects on this component (e.g., Sass et al., 2010; Palazova et al., 2013; Zhang et al., 2014).

#### LPP

LPP amplitude responded to emotional content, being larger for both positive and negative than for neutral words. This accords with a large body of literature on emotional word processing (see e.g., Citron, 2012 for an overview), and is seen particularly during active tasks, requiring attentive processing of emotional content (Schindler and Kissler, 2016). The u-shaped, arousal-driven effect of emotional content is in line with the motivated attention account which is generally influential in the emotional stimulus perception literature (Lang et al., 1997) and also described by Kuperman (2015) for word processing. Crucially, emotional LPP modulation further varied with mood induction in that LPP amplitude was particularly pronounced for the mood-incongruent word valence. This pattern is similar to what was observed for the lateral N1 and EPN effects and may be in line with the above mentioned alerting by interrupt account. Herring et al. (2011) investigating evaluative affective priming also found that the LPP, but not the N400, responded to the priming manipulation, with the response pattern indicating incongruity-sensitivity on the LPP. The present data extend this pattern from picture and word priming to the effect of experimentally induced moods across blocks of stimuli. Although we have not found language effects in the LPP, a recent EEG study (Kao and Zhang, 2020) points out differences in emotional speech processing between L1 and L2 exactly in the late ERP components—N400 and LPP in the auditory modality. This shows that that language effects for emotional meaning are also modality-related, and future studies need to account not only for mood but also for modality effects when examining how bilinguals process emotional language in their respective linguistic systems.

### Limitations and Open Questions

The present study provides evidence for very early effects of mood on lateralization of language processing in L1, as well as of mood on emotion word processing in general. It also replicates several established effects, providing good conceptual credibility for the present findings. Since several observed effects were found in consecutive time-windows, there is also good internal consistency in the data. However, our aim of characterizing the full processing timeline across several time-windows necessitated numerous statistical tests. Therefore, the present findings should be replicated in the future and, if possible, larger groups should be studied. In fact, some early interactions also suggested that early mood effects on L1 versus L2 processing might be valence-specific as we had originally hypothesized. With more experimental power, it should be possible to further specify the nature of these effects. Using different, possibly less related languages may provide a further means of replication, but also help reveal specific effects. Finally, directly contrasting word and sentence-level effects in the same participants would allow us to test whether temporal shifts occur depending on processing load. Early mood effects might be specific to word-level processing and later ones (e.g., in the N400 window) might be found in sentence-level studies.

## Summary

Overall, we found that moods started modifying emotional word content processing very early, already at N1. This early influence was stronger for happy mood, bigger for L1, relative to L2, and clearly lateralized: left-lateralized for L1 and right-sided, in tendency, for L2, demonstrating language-specific mood effects in the bilingual brain that call for further characterization. Importantly, we found mood-congruent effects in perceptual processes and mood-incongruent ERP amplification during higher order evaluative processing, indicating that the effect of mood on the neurophysiology of language is stage-specific, rather than general. This needs to be taken into account by future models incorporating mood as a context factor in language processing.

## Data Availability Statement

The raw data supporting the conclusions of this article will be made available by the authors, without undue reservation.

## Ethics Statement

Ethical review and approval was not required for the study on human participants in accordance with the local legislation and institutional requirements. The patients/participants provided their written informed consent to participate in this study.

## Author Contributions

JK and KB-D designed and setup the study and wrote the manuscript. JK analyzed the data. Both authors contributed to the article and approved the submitted version.

## Conflict of Interest

The authors declare that the research was conducted in the absence of any commercial or financial relationships that could be construed as a potential conflict of interest.

## Footnotes

1 www.neurobehavioralsystems.com

2 www.biosemi.com

3 www.biosemi.com/faq/cms&drl.htm

4 www.besa.de
